# Comprehensive analysis of ectopic pregnancy outcomes among Indian women

**DOI:** 10.6026/973206300223169

**Published:** 2026-05-31

**Authors:** Sabiha Islam, Salma Akter Munmun, Rowson Ara, Monowara Begum, Khodeza Khatun, Shah Aziz, Parveen Akhter Shamsunnahar

**Affiliations:** 1 Department of Obstetrics and Gynaecology, Bangladesh Medical University (BMU), Dhaka, Bangladesh

**Keywords:** Ectopic pregnancy, risk factors, clinical manifestations

## Abstract

Ectopic pregnancy (EP) is an emergency where an embryo implants outside the uterus, often in the fallopian tubes. Hence, this retrospective descriptive study was conducted at the Department of Obstetrics and Gynecology (indoor) in Bangabandhu Sheikh Mujib Medical University, Dhaka from January 2022 December 2022. In this investigation of 55 ectopic pregnancy cases, urine pregnancy tests and βHCG assays were used for provisional diagnosis, while ultrasound imaging confirmed it. A thorough risk factor assessment and prompt diagnosis are crucial for preserving fertility and reducing morbidity and mortality in ectopic pregnancies. Increased awareness and early detection of unruptured cases, especially in peripheral centers, can enable timely referral and treatment.

## Background:

Ectopic pregnancy is a prevalent acute gynecological emergency involving the implantation of an embryo outside the uterine cavity, most frequently within the fallopian tubes [1]. Normally, smooth muscle contractions and ciliary movements in the fallopian tubes facilitate the transport of the oocyte and embryo. However, damage to the fallopian tubes often due to inflammation can impair their function, leading to the retention of the oocyte or embryo [2]. Ectopic pregnancy (EP) ruptures are a major cause of maternal mortality during the first trimester, accounting for 9-14% of such deaths and comprising 5-10% of all pregnancy-related fatalities [3]. Various risk factors contribute to ectopic pregnancy, including a history of previous ectopic pregnancies, reproductive system infections, and multiple sexual partners, past miscarriages, use of assisted reproductive technologies, current use of an intrauterine contraceptive device (IUCD), previous cesarean sections (CS) and cigarette smoking at the time of conception [4]. Additional factors contributing to ectopic pregnancies include previous tubal surgeries, tubal sterilization, a history of abortions, advanced maternal age, parity and prior pelvic and abdominal surgeries [5, 6]. Tubal ectopic pregnancies (EPs) are the most prevalent type and are associated with significant maternal morbidity and mortality if they rupture [3]. In Western countries, the incidence of ruptured ectopic pregnancies is around 15% [7]. Diagnosing ectopic pregnancy poses a significant challenge for obstetricians and gynecologists due to its diverse and often ambiguous clinical manifestations. Symptoms can range from being asymptomatic to presenting as nonspecific issues like lower abdominal pain and vaginal bleeding. These symptoms frequently mimic those of other conditions, such as appendicitis, urinary stones, early pregnancy loss, trauma, or hemodynamic shock, making accurate diagnosis particularly complex [8]. In the first trimester, women presenting with this condition may have an ectopic pregnancy (EP) prevalence of up to 18% in emergency departments. This high rate can lead to frequent misdiagnoses, as it can be easily mistaken for other conditions with similar clinical presentations [9]. This frequently results in delayed diagnoses with severe consequences.

To achieve early detection, particularly in women of reproductive age, a high level of clinical suspicion is crucial. The current diagnostic standards involve ultrasound imaging either transvaginal (TVUS) or transabdominal (TAUS) and monitoring of β-human chorionic gonadotropin (β-hCG) levels. Timely and accurate diagnosis of ectopic pregnancy (EP) can significantly decrease maternal mortality rates [10]. Once an ectopic pregnancy (EP) is diagnosed, the treatment approach is tailored based on several factors, including the location of the EP, the stage of pregnancy and the size of the gestational sac. Treatment options generally fall into three categories: medical management, surgical intervention and expectant management. The choice among these approaches depends on the specific characteristics of the EP [10]. Surgical treatments include partial salpingectomy, which involves removing a section of the fallopian tube; total salpingectomy, which entails the complete removal of the fallopian tube; salpingo-oophorectomy, where both the affected tube and ovary are removed; and hysterectomy, a more extensive procedure involving the removal of the uterus in severe cases. On the other hand, medical management predominantly relies on methotrexate therapy. This approach is particularly effective for unruptured ectopic pregnancies and offers a non-invasive treatment option [11]. Therefore, it is of interest to report the frequency, risk factors, clinical manifestations and management strategies of ectopic pregnancy at a tertiary care facility.

## Methodology and Materials:

This retrospective descriptive study was conducted at the Department of Obstetrics and Gynecology (indoor) in Bangladesh Medical University (BMU), Dhaka from January 2022 December 2022. The study focused on analyzing cases of ectopic pregnancy that were managed at the hospital during this period. The hospital is a tertiary care centre that serves a diverse population, providing comprehensive obstetric and gynecologic services. The study included all women diagnosed with ectopic pregnancy and managed at BSMMU during the study period. A total of 55 cases were identified and included in the analysis.

## Inclusion criteria: 

All women aged more than or equal to 18 with a confirmed diagnosis of ectopic pregnancy were included in the study.

## Exclusion criteria:

There were no exclusion criteria for this study; all confirmed cases of ectopic pregnancy were analyzed.

## Data collection:

Data were collected from the hospital's electronic medical records system and included demographic details, clinical presentation, diagnostic modalities, management strategies and outcomes of the patients. The collected data points include age, gravidity and gestational age at diagnosis, history of previous ectopic pregnancy, risk factors and location of the ectopic pregnancy, diagnostic methods (ultrasound, β-hCG levels), treatment modality and any associated complications.

## Statistical analysis:

Data were entered and analyzed using Statistical Package for Social Science (SPSS version 26.0). Descriptive statistics were used to summarize patient demographics, clinical characteristics and treatment outcomes. Categorical variables were expressed as frequencies and percentages, while continuous variables were presented as means ± standard deviations.

## Results:

**Figure 1:**
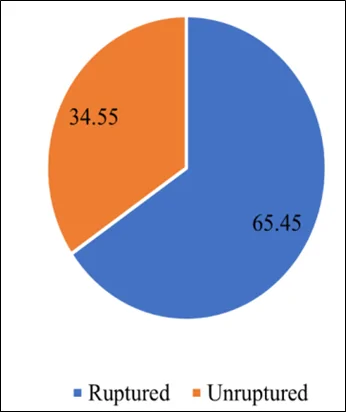
Type of ectopic pregnancy

In this investigation, we identified 55 cases of ectopic pregnancy. For provisional diagnosis, both urine pregnancy tests and βHCG assays were performed, while ultrasound imaging was instrumental in confirming the diagnosis of ectopic pregnancy. The gestational age of the cases varied from 4 to 10 weeks, with a mean gestational age of 6.51±1.13 weeks. The demographic overview presented in Table 1 reveals that the majority of participants fall within the 25-30 age range (49.09%), with a mean age of 27.41 years (SD ±3.52). Obstetric scores indicate that most participants are in their second pregnancy (47.27%), followed by those in their first (25.45%) and third or higher pregnancies (27.27%). Table 2 highlights the prevalence of various risk factors within the study population, with previous C-sections (27.27%) and pelvic surgeries (21.82%) being the most common. Other notable risk factors include previous abortions/MTPs (7.27%) and pelvic inflammatory disease (PID) (10.91%). Table 3 details the clinical characteristics observed, where the triad of symptoms (36.36%) is the most frequently reported presentation, followed by abdominal pain (25.45%) and shock (10.91%). Other symptoms include bleeding per vaginum (10.91%) and asymptomatic cases (9.09%). Figure 1 illustrates the classification of ectopic pregnancies. Among the cases, 36 (65.45%) were identified as ruptured ectopic pregnancies, while 19 (34.55%) were classified as unruptured. The majority of these cases were managed through surgical intervention.

**Figure 2:**
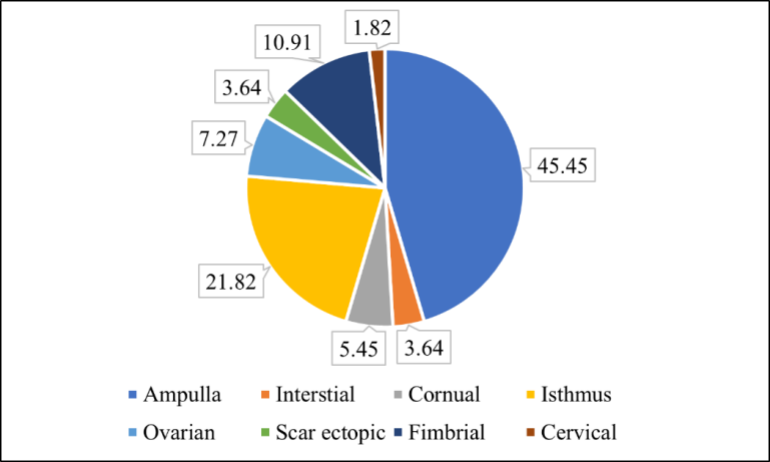
Site-specific occurrence of ectopic pregnancies

Table 4 provides a comprehensive overview of the treatment modalities administered, detailing their frequency and percentage. The most frequently employed interventions were total salpingectomy, accounting for 43.64% of cases and partial salpingectomy, which was utilized in 40.00% of instances. In contrast, salpingo-oophorectomy was used in only 5.45% of cases, while medical management and corneal resection each represented 3.64%. Hysterectomy and scar ectopic, each with a frequency of 1.82%, were the least common treatments. This distribution highlights the predominance of salpingectomy procedures in the management strategy. Figure 2 illustrates the site-specific distribution of ectopic pregnancies. The majority of ectopic pregnancies occurred in the ampulla (45.45%), followed by the isthmus (21.82%) and fimbrial sites (10.91%). Other sites included ovarian (7.27%), cornual (5.45%), interstial (3.64%), scar ectopic (3.64%) and cervical locations (1.82%). Table 5 illustrates the patterns of morbidity and mortality among study patients, highlighting various outcomes and their frequencies. Notably, nearly half of the patients (49.09%) required admission to the High Dependency Unit (HDU), while 18.18% underwent blood transfusions. Intensive Care Unit (ICU) admission and prolonged hospital stay each affected 14.55% of patients. Acute renal failure and mortality were relatively rare, affecting 1.82% of the patients each.

## Discussion:

The earliest documented case of ectopic pregnancy, attributed to Al-Zahrawi in the 11th century, identified it as a cause of maternal mortality. Remarkably, it remains a leading cause of maternal death in early pregnancy today [12, 13]. In this study, the most affected age group was women between 18 and 30 years and this finding is consistent with a study by Naznin *et al.* which reported the highest incidence of ectopic pregnancies in women aged 26-30 years [14]. Conversely, a study by Battu *et al.* in the USA showed that the incidence of ectopic pregnancy increases with age [15], likely due to early marriage and childbearing practices in Bangladesh, where fewer pregnancies occur after the age of 30. The mean gestational age in this study was 6.51±1.13 weeks, which aligns with findings by Zhang *et al.* [16]. Studies differ regarding the association between gravida and ectopic pregnancy, with some reporting the highest incidence among primigravida, while others, show third or fourth gravida as most affected [14, 17]. In our study, parous women, especially those with one previous child, were more frequently diagnosed with ectopic pregnancy. Upon assessing risk factors, previous pelvic surgery emerged as the most common, particularly previous caesarean sections, a finding consistent with studies by Zhang *et al.* and Talukder *et al.* [16, 18]. Pelvic inflammatory disease (PID) is another known risk factor, increasing the risk of ectopic pregnancy by 3.3 to 6 times due to infections like gonococci and chlamydia [19]. In our study, 10.91% of women had a history of PID. Similarly, Chowdary & Panda found the incidence to be 10-11% [19]. Mahajan *et al.* also identified a strong link between chlamydial infection and tubal pregnancy [20]. Changes in sexual activity can lead to pelvic inflammation and tubal damage, particularly in younger women, thereby increasing the risk of ectopic pregnancies [19]. Intrauterine device (IUD) use was observed in 7.27% of our patients. Kumari *et al.* reported a similar incidence, supporting the idea thatpregnancies occurring with an IUD in place are likely ectopic [21]. Previous abortions are also a significant risk factor for ectopic pregnancy, cited in 17-31% of cases across various studies [17, 22], though only 7.27% of our patients had a history of abortion. Advancements in reproductive technology, including ovulation induction drugs, have led to an increase in ectopic pregnancies, with infertility treatments contributing to 1.82% of cases in this study. This reflects a broader trend of increasing ectopic pregnancy rates associated with assisted reproductive technology [18, 22]. A history of previous ectopic pregnancy was found in 3.64% of cases, consistent with other studies, while risk factors remained unidentified in 20% of our cases. The classic triad of symptoms was present in 36.36% of cases in this study, which is higher than the 27.7% and 31.9% reported by Alam et al. and Talukder et al. respectively [17, 18]. Abdominal pain was the most common symptom, observed in 25.45% of cases, though it is often nonspecific. Vaginal bleeding was seen in 10.91% of patients, a symptom that, on its own, is insufficient for diagnosis. Amenorrhea, however, raised early suspicion of pregnancy and aided in further diagnostic work. Due to the late referral of cases, 65.45% of our patients presented with ruptured ectopic pregnancies, a rate lower than the 82.5% reported by Gilmore & Hardman [23]. This highlights the delays in early diagnosis and referral. However, 33.55% of our cases were diagnosed with unruptured ectopic pregnancies [24]. The most common site of ectopic implantation was the ampulla (45.45%), consistent with other studies. Less common locations included cervical, scar and interstitial ectopic pregnancies, with extra-tubal ectopic pregnancies being rare. Management of ectopic pregnancies focuses on either life-saving interventions or preserving fertility [26,26]. In this study, 96.36% of patients underwent surgical management, with laparotomy being the most frequent procedure. Salpingectomy was performed in 83.64% of cases, while salpingo-oophorectomy was necessary in 5.45%. Other studies have reported similar rates of open salpingectomy, ranging from 70% to 90% by Naznin *et al.* and Talukder *et al.* [14, 18]. Nonsurgical management, including medical and expectant approaches, was successful in 3.64% of cases, comparable to previous study by Khandkar *et al.* and Xiao *et al.* which reported success rates of 1.75% and 2.5%, respectively [27, 28]. In this study, 18.18% of patients required blood transfusions, a significantly lower rate than the 84.61% reported by Zhang *et al.* [16]. The high rate of ruptured ectopic pregnancies in our study may explain the need for blood transfusions.

## Limitations:

Of the study include a small sample size and a single-center design, which limit the generalizability of the findings. The retrospective nature may introduce biases and the lack of long-term follow-up prevents assessment of outcomes such as future fertility. The study primarily focuses on surgical interventions, providing limited insight into non-surgical management. Exploration of risk factors is restricted and the absence of a control group limits the ability to evaluate relative risks. It is recommended to carefully assess patients for prior obstetric history and pelvic risk factors, as these were common in the study population. Early diagnosis and appropriate selection of surgical or medical management strategies may help reduce morbidity and improve patient outcomes.

## Conclusion:

A thorough assessment of risk factors and prompt diagnosis are essential for safeguarding fertility and reducing both morbidity and mortality. Data shows that most cases presented in a ruptured state, making conservative management unfeasible. Increased awareness of the contributing factors could facilitate earlier detection of more unruptured cases, particularly in peripheral centers, enabling timely referral and intervention.

## Figures and Tables

**Table 1:** Demographic overview of the participants

**Variable**	**Frequency (n)**	**Percentage (%)**
Age range (in years)		
18-25	19	34.55
25-30	27	49.09
> 30	9	16.36
Mean±SD		27.41±3.52
Obstetric score (gravida)		
G1	14	25.45
G2	26	47.27
G3	11	20
Above G3	4	7.27

**Table 2:** Prevalence of risk factors among the study population

**Risk factors**	**Frequency (n)**	**Percentage (%)**
Previous abortion/ MTP	4	7.27
ART	1	1.82
PID	6	10.91
IUCD	4	7.27
Pelvic surgery	12	21.82
Previous C-section	15	27.27
Previous ectopic	2	3.64
No risk	11	20

**Table 3:** Clinical characteristics of the study population

**Clinical presentation**	**Frequency (n)**	**Percentage (%)**
Triad	20	36.36
Shock	6	10.91
Pain abdomen	14	25.45
Bleeding PV	6	10.91
Amenorrhea	3	5.45
Asymptomatic	5	9.09
Acute abdomen	1	1.82

**Table 4:** Overview of various treatment modalities

**Intervention**	**Frequency (n)**	**Percentage (%)**
Partial salpingectomy	22	40
Total salpingectomy	24	43.64
Salpingo-oophorectomy	3	5.45
Medical management	2	3.64
Hysterectomy	1	1.82
Corneal resection	2	3.64
Scar ectopic	1	1.82

**Table 5:** Patterns of morbidity and mortality in study patients

**Morbidity and mortality**	**Frequency (n)**	**Percentage (%)**
HDU admission	27	49.09
Blood transfusion	10	18.18
ICU admission	8	14.55
Prolong stay in hospital	8	14.55
Acute renal failure	1	1.82
Mortality	1	1.82

## References

[1] Kalyankar V (2022). New Indian J Obgyn..

[2] Barton B (2020). Reproduction..

[3] Houser M (2022). Emerg Radiol..

[4] Singh S (2025). J Family Med Prim Care..

[5] Brim A.C.S (2025). Int J Gynaecol Obstet..

[6] https://emedicine.medscape.com/article/2041923-overview?utm..

[7] Dvash S (2021). Eur J Obstet Gynecol Reprod Biol..

[8] Hendriks E (2020). Am Fam Physician..

[9] Khatun M.H.A (2020). J Dhaka Natl Med Coll Hosp..

[10] Mullany K (2023). Womens Health (Lond)..

[1] https://rcog-irc-bd.org/bangladesh-focused-guideline-ectopic-pregnancy-adapted-from-rcog-nice-includes-csp-cervical-pregnancy/?utm..

[12] Chong K.Y (2024). Nat Rev Dis Primers..

[13] Qian H (2025). Eur J Obstet Gynecol Reprod Biol..

[14] Naznin N (2025). J Curr Adv Med Res..

[15] Battu G (2023). Indian J Obstet Gynecol Res..

[16] Zhang S (2023). PLoS One..

[17] Alam N (2023). Front Public Health..

[18] Talukder A (2023). J Med Sci Health..

[19] Chowdary P.S, Panda S. (2023). Int J Clin Obstet Gynaecol..

[20] Mahajan N (2021). Iberoam J Med..

[21] Kumari P (2025). J Family Med Prim Care..

[22] Andola S (2021). J Family Med Prim Care..

[23] Gilmore K.L, Hardman S.M. (2021). BMJ Sex Reprod Health..

[24] Murugesan A (2016). Int J Reprod Contracept Obstet Gynecol..

[25] Sefogah P.E (2022). Obstet Gynecol Int..

[26] Choudhary S (2025). Int J Reprod Contracept Obstet Gynecol..

[27] Khandkar SA (2024). Int J Reprod Contracept Obstet Gynecol..

[28] Xiao C (2021). Medicine (Baltimore)..

